# Impact of the relative dose intensity on survival of patients with high‐risk myelodysplastic syndromes treated with Azacitidine

**DOI:** 10.1002/cam4.2121

**Published:** 2019-04-16

**Authors:** Kamel Laribi, Delphine Bolle, Mustafa Alani, Habib Ghnaya, Anne Besançon, Jonathan Farhi, Kayane Mheidly, Nathalie Denizon, Alix Baugier de Materre

**Affiliations:** ^1^ Department of Hematology Centre Hospitalier Le Mans Le Mans France; ^2^ Department of Pharmacy Centre Hospitalier Le Mans Le Mans France; ^3^ Université Paris-Saclay, Université Paris-Sud CESP (Center for Research in Epidemiology and Population Health), Inserm, Team Cancer and Environment Villejuif France

**Keywords:** Azacitidine, dose intensity, myelodisplastic syndromes

## Abstract

We performed a retrospective analysis of 93 myelodysplastic syndromes (MDS) patients with intermediate 2 or high‐risk IPSS score to study the impact of Azacitidine (AZA) relative dose intensity (RDI) <80% on the overall survival (OS). There were 51.6% of patients who had full dose and 48.4% had dose reduction or delayed with a RDI <80%. Nineteen patients (20.4%) had RDI <80% before getting objective response. Overall and progression‐free survivals (OS, PFS) probabilities for the whole population were 58% (95% CI: 48‐69) and 47% (95% CI: 38‐58) at 1 year; 35% (95% CI: 26‐47) and 31% (95% CI: 23‐43) at two years, respectively. When analyzing the outcomes according to the response to AZA, median OS was 32 months (range: 26‐55) for responders and 8 months (range: 7‐12) for nonresponders, with a respective 1‐year and 2‐year OS probabilities of 91% vs 28% and 66% vs 6%, respectively (*P* < 0.001). Interestingly, there was no impact of dose reduction on OS nor on PFS, however, when analyzing the timing of dose reduction as time‐dependent variable, we found that patients who had dose reduction before achieving the objective response, had significantly lower OS (*P* = 0.02) and PFS (*P* = 0.01) compared to patients who had dose reduction after achieving the objective response. In multivariate analysis, acute myeloid leukemia with 21%‐30% blasts in BM and poor and very poor karyotype significantly impacted OS, (HR = 2.09, 95% CI: 1.27‐3.44, *P *= 0.004, and HR = 2.73, 95% CI: 1.6‐4.6, *P *< 0.001 respectively), as well as PFS (HR = 1.84, 95% CI: 1.07‐3.17, *P *= 0.028, and HR = 3.03, 95% CI: 1.7‐5.39, *P *< 0.001, respectively).

## 1 INTRODUCTION

Myelodysplastic syndromes (MDS) are a group of bone marrow stem cell disorders characterized by ineffective hematopoiesis leading to blood cytopenias and a high incidence of progression to acute myeloid leukemia (AML) occurring in one‐third of cases.^1^, ^2^, ^3^, ^4^ Irregular DNA hypermethylation is a hallmark of MDS^5^, ^6^, ^7^ and among hypomethylating agents, azacitidine (AZA) at a standard daily dose of 75 mg/m^2^ for 7 days spaced every 4 weeks, significantly reduces transfusion dependence, decreases the risk of transformation to AML, and improves quality of life (QOL) in high‐risk MDS (ie intermediate‐2 or high IPSS risk MDS).^8^, ^9^, ^10^

In routine clinical use, the dosage regimen of AZA could be adapted to the care environment of each treating center: 7 consecutively days or 5 days of treatment, 2 days off, and 2 days of treatment (5‐2‐2 regimen) are the most used. However, dose reduced regimen as a 5 days schedule or delayed treatment is also used in certain circumstances related mainly to the tolerance of the drug or a myelosuppression as a result of MDS.

In a phase III AZA‐001 trial^11^, 21% of cycles were longer than 35 days, and in the Spanish registry,^12^ 57.5% of patients received reduced dose regimen with a total number of days less than 7 days.

Few studies have assessed the efficacy and safety of alternatives dosing schedule of AZA like a 5‐day schedule of subcutaneous or intravenous form or 5‐day dose‐intensified regimen and results from using these schedules are still not homogenous. Furthermore, the population analyzed was generally heterogeneous with a significant portion of lower‐risk MDS patients.^13^, ^14^, ^15^, ^16^, ^17^

The GFM group reported a large series (n = 282) of patients treated in the compassionate, patient‐named program of AZA in IPSS intermediate‐2 or high‐risk MDS and AML with ≤30% marrow blasts and showed that reduction of AZA schedule in 28% of patients did not significantly influence overall survival (OS) patients (median, 10.3 vs 14.3 months; *P* = 0.10).^18^

On the other hand, no prospective study has compared the standard AZA regimen to another dose schedule in responding patients or the analysis of a maintenance treatment with attenuated doses in responders comparatively to the standard regimen.

The primary endpoint of the present study is to assess the impact of the dose reduction and/or dose delay on the outcome of patients with MDS treated with AZA at our institution. The secondary endpoints were the evaluation of the efficacy of AZA in patients with high‐risk MDS and AML with 21%‐30% BM blasts according to MDS‐WHO criteria, safety, OS, and factors impacting on OS.

## 2 MATERIAL AND METHODS

### 2.1 Patients

We retrospectively analyzed the efficacy of AZA in patients older than 18 years of age with intermediate‐2 or high‐risk MDS (as defined by the WHO‐ 2008 criteria),^19^ according to the IPSS,^8^ nonproliferative chronic myelomonocytic leukemia with 10%‐19% marrow blasts (CMML‐2 with WBC<13G/L) or acute myelogenous leukemia (AML) with 20%‐30% blasts and multi‐lineage dysplasia, treated in the front‐line setting in the Hematology Department of Le Mans Hospital center between November 2008 and February 2018. This retrospective study was approved by the local ethics committee.

### 2.2 Treatment, dose relative intensity, and assessment of response

Azacitidine was administered subcutaneously according to the 5 days regimen of treatment, 2 days off and 2 days on treatment (5‐2‐2). Dose reduction of AZA including a 5 days regimen or delays to every 5 weeks were considered before hematological toxicity or nonhematologic‐related complications, and could be done for some patients depending on tolerance, compliance, and psychological tolerance after the achievement of best response objective. The dose intensity is the amount of AZA administered per unit time. The relative dose intensity (RDI) is the percentage of the dose received by the patient on the dose that theoretically should have administered.

Thus, a patient receiving a 5 days regimen of AZA every 4 weeks received RDI of 5/7 (71%) of AZA, and the one who receives 7 days every 5 weeks received in 6 months 83% of the RDI. We calculated the RDI of AZA for all patients and compared a group of patients who received RDI ≥80% of AZA with a group of patients who received RDI <80% of AZA.

Cytogenetic abnormalities were classified according to International System for Human Cytogenetic Nomenclature criteria,^20^, ^21^ IPSS^8^ criteria, and IPSS‐R criteria.^22^ Responses to treatment and disease status were evaluated according to IWG‐MDS‐ 2006^23^ response criteria. The first evaluation of response was carried out after the fourth cycle of AZA. Progression defining events were death due to any reason, disease progression, disease relapse after response, and/or new cytogenetic aberration/clonal evolution.

### 2.3 Statistical analysis

All time‐to‐event analyses were performed from AZA initiation date using the Kaplan‐Meier method and the log‐rank test. OS was calculated from the start of AZA therapy until death from any cause, progression‐free survival (PFS) was defined as the time from AZA initiation to the time of first event (progression of disease or death). Clinical parameters that were found to have significant effect on survival in univariate analysis (those covariates with *P* values < 0.1) were selected through a stepwise algorithm and then reevaluated in a multivariate model with the addition of variables with clinical relevance (such as RDI <80 used as time‐dependent variable). Statistical analysis was performed using R statistical software (v3.5.1).

## 3 RESULTS

### 3.1 Patient characteristics

This retrospective analysis included 93 patients who received AZA in the front‐line setting at the Hematology Department of Le Mans Hospital center between November 2008 and February 2018. The median age was 77 years (range: 56‐89), the majority of the patients (59%) had more than 75 years, with a male predominance (sex ratio: 1.44). Sixty‐one patients (66%) had 2 or 3 cytopenias and forty‐five (48%) had transfusion dependence represented by the transfusion of ≥4 RBC/unit/8 weeks, or platelets, before the start of AZA regimen. Twenty‐two patients (24%) had AML with 20%‐30% blasts and multi‐lineage dysplasia. Cytogenetic analysis according to IPSS score was favorable, intermediate, or poor risk in 49%, 15%, and 29% of patients, respectively. The IPSS risk score was intermediate risk 2 in 57 patients (61%), and high risk in 31 patients (33%). Patient characteristics are shown in Table 1.

**Table 1:** Demographic and baseline characteristics of AZA‐treated patients

Characteristics	N (%)
Age	
Median (range)	77 (56‐89)
>75 years	55 (59%)
≤75 years	38 (41%)
Gender (male)	55 (59%)
PS ECOG	
0‐1	61 (66%)
2‐3	26 (28%)
Missing	6 (6%)
Albumin (g/L)	
≤35	34 (37%)
>35	47 (50%)
Missing	12 (13%)
LDH level (UI/L)	
≤250	40 (43%)
>250	42 (45%)
Missing	11 (12%)
Ferritin level (ng/mL)	
≥1000	19 (21%)
<1000	44 (47%)
Missing	30 (32%)
Blasts	
Median (range)	13% (4‐30)
Cytopenias	
0‐1	32 (34%)
2‐3	61 (66%)
WHO diagnosis	
CRMD	2 (2%)
AREB1	10 (11%)
AREB2	44 (47%)
LMMC2	15 (16%)
AML	22 (24%)
Transfusion dependence	
Yes	45 (48%)
Cytogenetic risk (IPSS)	
Favorable	46 (49%)
Intermediate	14 (15%)
Poor	27 (29%)
Missing	6 (7%)
Cytogenetic risk (IPSS‐R)	
Very good	6 (6%)
Good	46 (50%)
Intermediate	10 (11%)
Poor	5 (5%)
Very poor	20 (22%)
Missing	6 (6%)
IPSS risk score	
Intermediate risk 2 (1.5‐2)	57 (61.3%)
High risk (2.5‐3)	31 (33.3%)
Missing	5 (5.4%)
IPSS‐R risk score	
Intermediate > 3‐4.5	22 (24%)
High > 4.5‐6	35 (38%)
Very high > 6	30 (32%)
Missing	6 (6%)

AML, acute myeloid leukemia; AZA, Azacitidine.

### 3.2 Relative dose intensity

Forty‐eight patients (52%) had a full dose and 45 (48%) had dose reduction or delayed with a RDI <80%. Twenty‐eight patients (30%) received 5 days regimen, 22 (24%) patients were treated with cycles of more than 35 days, and five (5%) patients had received the two procedures. Patients with cycle delays or dose reductions received a median of 15 cycles (2‐68) while those without delays or dose reductions received a median of 6 cycles (1‐39). Twenty‐six patients (28%) had dose reduction and/or dose delayed with RDI <80% after achieving best objective response and 19 (20%) had a RDI <80% before reaching the best objective response. Twenty‐nine (31%) patients had a RDI <80% less before reaching cycle 12 of AZA and 16 (17%) patients had their dose reduced at cycle 12 or later (Table 2).

**Table 2:** Relative dose Intensity of AZA < 80%

Patients with AZA‐RDI < 80%	N	%
Total	45	48
5 days regimen (A)	28	30
5‐2‐2 regimen every 5 weeks or >5 weeks	22	24
Two procedures (A) + (B)	5	5
Patients achieved BOR	26	28
Patients did not achieve BOR	19	20
Patients who received less than 12 cycles of AZA	29	31
Patients who received 12 cycles of AZA or plus	16	17

AZA, Azacitidine; BOR, best objectives response; RDI, relative dose intensity.

### 3.3 Response to treatment

Median time from first diagnosis to treatment start with AZA was 1.1 months (range: 0.2‐8.9). The median time from the start of AZA to treatment response was 4 months (range: 0.3‐8.97). The median number of AZA cycles was 9 (1‐68). Best hematological response according to IWG 2006 criteria was achieved in 53 patients (57%) after a median time of 4 months. According to IWG –MDS response criteria 2006, Overall response defined as CR, marrow CR, PR, and HI, was documented in 57% of patients (CR: 17.2%, marrow CR: 20.4%, PR: 9.7%, stable disease (SD) with hematological improvement: 9.7%) (Table 3). Forty‐two percent of patients were nonresponders including 25% of patients who achieved SD without hematologic improvement and 17% of patients who experienced treatment failure after AZA.

**Table 3:** Response to treatment according to IWG‐MDS response criteria 2006

	Median (range)	N	(%)
Complete response		16	17.2
Partial response		9	9.7
Bone marrow remission		19	20.4
Stable disease		32	34.4
with HI		9	9.7
without HI		23	24.7
Progression		16	17.2
missing		1	1.1
Hematological improvement		35	32.5
Platelet (HI‐P)		25/60	41.6
Erythroid (HI‐E)		20/59	33.9
Neutrophil (HI‐N)		14/56	25
Transfusion independence after AZA			
Yes		14/45	31.1
No		31/45	68.9
Duration of AZA (No. of cycles)	9 (1‐68)		

AZA, Azacitidine; HI: hematological improvement, MDS, myelodysplastic syndromes.

Hematological improvement was obtained in 35 patients (32.5%), with a better response in platelet lineage (42.6%) and erythroid lineage (33.9%). Neutrophil improvement was shown in 25% of patients. Among the 45 patients who had transfusion dependence before the start of AZA, 14 patients (31.1%) had transfusion independence at least 8 weeks according to IWG2006‐MDS response criteria. Response details are summarized in Table 3.

### 3.4 Causes of AZA discontinuation and death

At the last follow‐up, 82 patients (88.2%) discontinued AZA. The causes of discontinuation were a lack of significant improvement or disease progression which involved in 16 patients (17.2%), relapse in 28 patients (30.1%), and alteration of general conditions in 10 patients (10.7%). Infections occurred in 17 patients (18.3%), hemorrhage in four patients (4.3%), heart failure in three patients (3.2%), and second cancer occurred in one patient (1.1%).Three patients (3.2%) discontinued treatment due to personal choice.

At the time of analysis, 17 patients (18.3%) were still alive, among them 11 (11.8%) were still treated with AZA. Seventy‐six patients (81.7%) have died, among them fifty‐two (55.9%) after discontinuation of AZA, all of complications related to disease progression.

### 3.5 Survival outcomes

**Figure 1:**
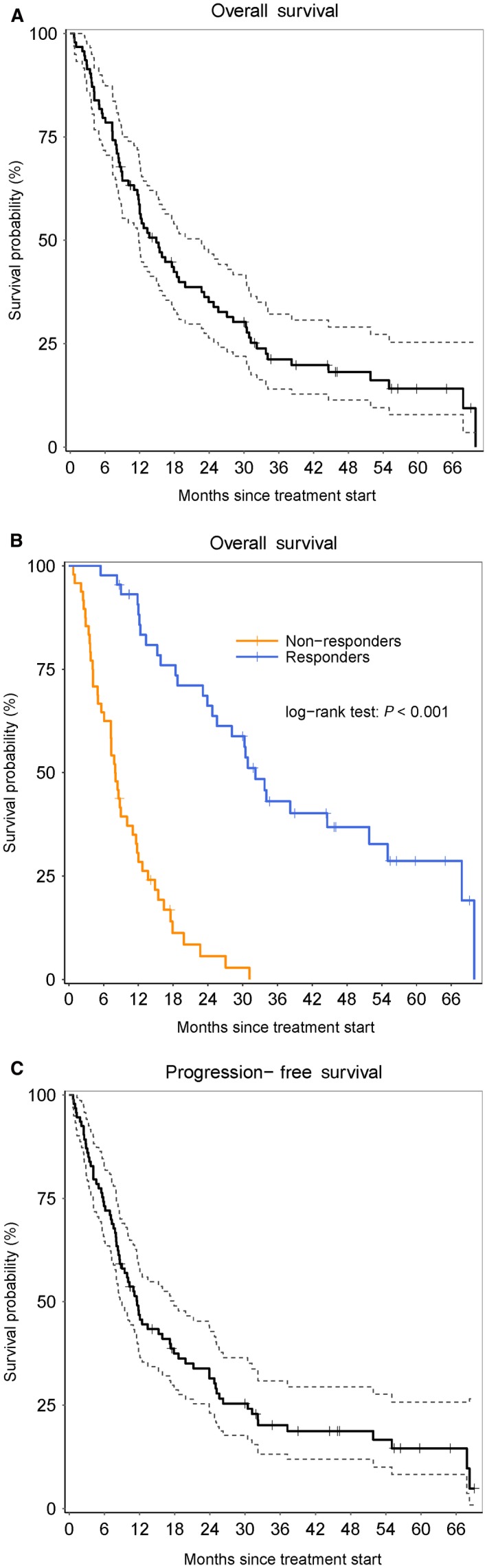
A, Overall survival for the whole population. B, Overall survival according to response after azacitidine (AZA). C, Progression‐free survival for the whole population

After a median follow‐up of 12 months (1‐71), a total of 76 events occurred, 39 relapse and 37 deaths without relapse, the median OS and PFS for the whole population were 15 months (range: 1‐71) and 12 months (range: 1‐70), respectively. OS and PFS probabilities for the whole population were 58% (95% CI: 48‐69) and 47% (95% CI: 38‐58) at one year; 35% (95% CI: 26‐47) and 31% (95% CI: 23‐43) at 2 years, respectively (Figure 1A,C). When analyzing the outcomes according to response to AZA, median OS was 32 months (range: 26‐55) for responders (defined as CR/marrow CR/PR/HI) and 8 months (range: 1‐12) for nonresponders, with a respective 1‐year and 2‐year OS probabilities of 91% vs 28% and 66% vs 6%, respectively (*P* < 0.001) (Figure 1B). A total of 40 patients had transformation to AML after a median time of 11 months (range: 1‐68). The median response duration was 3.9 months.

We tested in univariate analysis, the variables that could impact outcome after AZA were as follows: age, gender, ferritin level, LDH level, albumin level, number of cytopenias, transfusion dependence, AML, poor karyotype (IPSS score), poor and very poor karyotype (IPSS‐R score), high‐risk IPSS score, high and very high‐risk IPSS‐R score, RDI <80% before and after getting BOR. This univariate analysis showed a significant negative impact of number cytopenia, transfusion dependence, AML, poor karyotype (IPSS score), poor and very poor karyotype (IPSS‐R score), and high IPSS score on both OS and PFS (Table 4). Interestingly, there was no impact of dose reduction on OS nor on PFS, however, when analyzing the timing of dose reduction as time‐dependent variable, we found that patients who had dose reduction before achieving the objective response, had significantly lower OS (*P* = 0.02) and PFS (*P* = 0.01) compared to patients who had dose reduction after achieving the objective response. Significant factors in univariate analysis were studied in a multivariate model and the only two factors that were found to impact OS were AML with 21%‐30% blasts in BM (HR = 2.09, 95% CI: 1.27‐3.44, *P* = 0.004) and poor and very poor karyotype according to IPSS‐R (HR = 2.73, 95% CI: 1.6‐4.6, *P* < 0.001), as well as the same variables for PFS, with HR = 1.84, 95% CI: 1.07‐3.17, *P* = 0.028, and HR = 3.03, 95% CI: 1.7‐5.39, *P* < 0.001 respectively (Table 5).

**Table 4:** Univariate analysis on OS and PFS

Variable	OS			PFS		
	HR	95% CI	Cox *P*‐value	HR	95% CI	Cox *P*‐value
Cytopenias: 2‐3	1.66	[1.04‐2.63]	0.032	1.64	[1.03‐2.60]	0.036
Transfusion dependence	1.78	[1.12‐2.82]	0.014	1.68	[1.06‐2.65]	0.027
AML with 21%‐30% blasts in the bone marrow	2.01	[1.22‐3.31]	0.006	1.83	[1.11‐3.00]	0.017
Poor karyotype (IPSS score)	2.73	[1.49‐4.99]	0.001	2.92	[1.62‐5.26]	<0.001
Poor and very poor karyotype (IPSS‐R score)	2.86	[1.60‐5.09]	<0.001	2.87	[1.66‐4.94]	<0.001
High IPSS score	2.62	[1.83‐3.76]	<0.001	2.47	[1.71‐3.58]	<0.001
<80% RDI (time‐dependent)	0.732	[0.44‐1.16]	0.182	1.02	[0.69‐1.51]	0.911

AML, acute myeloid leukemia; OS, Overall survivals; PFS, progression‐free survivals; RDI, relative dose intensity.

**Table 5:** Multivariate analysis on OS and PFS

Variable	OS			PFS		
	HR	95% CI	Cox *P*‐value	HR	95% CI	Cox *P*‐value
AML with 21%‐30% blasts in the bone marrow	2.09	[1.27‐3.44]	0.004	1.84	[1.07‐3.17]	0.028
Poor and very poor karyotype (IPSS‐R score)	2.73	[1.60‐4.65]	<0.001	3.03	[1.70‐5.39]	<0.001
<80% RDI (time‐dependent)	0.77	[0.48‐1.24]	0.282	0.87	[0.54‐1.40]	0.558

AML, acute myeloid leukemia; OS, Overall survivals; PFS, progression‐free survivals; RDI, relative dose intensity.

## 4 DISCUSSION

The approved AZA dosing schedule is 75 mg/m^2^/day on days 1‐7 of each 28‐day treatment cycle (AZA 7). However, the consecutively 7 day regimen is most often not applied over the entire duration of treatment for multiple reasons.

In a prospective, longitudinal, multicenter US patients registry from community‐based hematology clinics (n = 331), it has been shown that only 17% of patients treated with AZA received the FDA‐approved continuous 7‐day dosing schedule; 51% received <7 days; 30% received 7 days with pauses; and 2% >7 days.^24^

In the Spanish compassionate use registry, 200 patients with either a confirmed diagnosis of WHO‐defined MDS, or de novo or secondary AML according to WHO criteria with 20%‐30% bone marrow blasts were treated with AZA: Among them 66 (33.0%) received AZA 5; 56 (28.0%) received AZA 5‐2‐2; and 78 (39.0%) received AZA 7 consecutively days regimen. ORRs appeared higher with the AZA 5‐2‐2 dosing schedule (67.9%) compared with AZA 7 (51.3%) and AZA 5 (39.4%), (overall, *P* = 0.0094), but there was no impact on survival. Otherwise, 58.5% of patients included in the study presented IPSS Low‐ or Int‐1‐risk MDS.^16^

Recently, an update of data registry of the GESMD between 2000 and 2013 included 251 patients with higher risk MDS patients (defined by an IPSS risk score of 1.5 or more) treated with AZA. Dosing schedule was available in 179 patients (71%). Seventy‐six patients received a standard 7‐day regimen (42.5%), and the remaining 103 (57.5%) were treated with less intensive dosing. There were no differences in survival between these two dosing schedules (*P* = 0.12).^12^

The MD Anderson group reported a retrospective pooled analysis of 2 decitabine, another HMA, clinical trials in 182 patients with de novo or secondary MDS, either of intermediate or high risk or of any French‐American‐British subtype and showed that patients who had dose modifications, cycle delays or dose reductions, had significantly higher ORRs compared with those who had none of these, but median OS values were similar to those of patients who had neither. Otherwise, patients with cycle delays or dose reductions received a median of six cycles of decitabine compared with those without cycle delays or dose reductions who received a median of two cycles.^25^

The MD Anderson group also studied the impact of RDI in patients with intermediate or high‐risk IPSS‐MDS or CMML treated with decitabine, in a monocentric retrospective series and showed OS rates significantly higher for patients who had dose reduction/or dose delayed after getting best objective response compared to those who had dose reduction/or dose delayed prior to best objective response or those with no dose reduction/or dose delayed.^26^

This could be explained by a longer exposure to treatment over time in patients with delayed/dose reduction compared to neither who received fewer cycles of AZA^25^ and the need for an initial intensity dose before obtaining a response to treatment and then maintaining of a certain degree of impregnation of HMA. In this line, it was shown that a low dose but high‐dose intensity schedule of decitabine at 20 mg/m^2^ intravenously daily over 1 hour for 5 consecutive days, every 4 week, optimizes hypo methylation induction, as well as clinical results based on IWG criteria, compared to the 10 mg/m^2^ intravenously over 1 hour daily for 10 days or 20 mg/m^2^ subcutaneously daily for 5 days regimens.^27^

The impact of the time of dose reduction on survival, or the maintenance therapy with reduced dose in patients who achieved best responses was not reported in patients treated with AZA. This is particularly important in MDS since patients are expected to receive long‐term treatment which can last several years, particularly in responders, but that can be a source of psychological intolerance, fatigue related to incessant trips to the hospital, alteration of QOL, and decision of discontinuation of AZA‐treatment, while it was demonstrated that additional cycles of AZA therapy have improved the quality of response in patients with higher‐risk MDS^28^ and a median of 9 cycles of AZA was previously showed to be involved with prolonged OS versus conventional care.^11^

The effect of dose relative intensity was largely unknown and to the best of our knowledge, this is the first report to address in depth the effect of dose relative intensity on patients' outcomes with high‐risk MDS treated with AZA. Nevertheless, this study has tried to provide answers to a question ever raised which is whether yes or no, responding patients beyond a certain time if they still need the same dose of AZA.

In this present study with the limitations related to its retrospective nature, we showed that 51.6% of patients had a full dose and 48.4% a dose reduction or delayed dose with a RDI <80%. Nineteen patients (20.4%) had RDI <80% before getting BOR, result of hematological toxicity or nonhematologic‐related complications that would lead the treating physician to reduce the dose(s) of AZA or delay subsequent doses. Twenty‐six patients (27.9%) expected dose reduction/or dose delayed after getting best objective response setting and 16 (17.2%) had their dose reduced at cycle 12 or after, for compliance or psychological tolerance.

An important finding in our study is that patients who had treatment dose reduced before achieving the objective response had significantly lower OS and PFS compared to those who had dose reduction after achieving response, this should be taken in high consideration when proceeding to dose reduction. Prospective evaluation of an approach conceiving a loading dose for induction of a best objective response followed by a maintenance schedule is to be considered for patients with MDS treated with AZA and HMA in general. In Japan, a prospective phase III trial (JALSG MDS212: UMIN000009633) that compares AZA: 75 mg/m^2^, 5 days regimen and AZA 75 mg/m^2^, 7 days regimen in high‐risk MDS patients, with OS as the end point, is ongoing. This study could provide valuable follow‐up information on the findings of our study.
